# Melatonin Attenuates Upregulation of *Duox1,* and *Duox2* and Protects against Lung Injury following Chest Irradiation in Rats

**DOI:** 10.22074/cellj.2019.6207

**Published:** 2019-06-15

**Authors:** Akbar Aliasgharzadeh, Bagher Farhood, Peyman Amini, Hana Saffar, Elahe Motevaseli, Saeed Rezapoor, Farzad Nouruzi, Dheyauldeen Shabeeb, Ahmed Eleojo Musa, Mehran Mohseni, Habiballah Moradi, Masoud Najafi

**Affiliations:** 1 Departments of Medical Physics and Radiology, Faculty of Paramedical Sciences, Kashan University of Medical Sciences, Kashan, Iran; 2 Department of Radiology, Faculty of Paramedical, Tehran University of Medical Sciences, Tehran, Iran; 3 Clinical and Anatomical Pathologist at Tehran University of Medical Science, Imam Khomeini Hospital Complex, Tehran, Iran; 4 Department of Molecular Medicine, School of Advanced Technologies in Medicine, Tehran University of Medical Sciences, Tehran, Iran; 5 Department of Medical Radiation Engineering, Science and Research Branch, Islamic Azad University, Tehran, Iran; 6 Department of Medical Physics and Biomedical Engineering, Faculty of Medicine, Tehran University of Medical Sciences (International Campus), Tehran, Iran; 7 Department of Physiology, College of Medicine, University of Misan, Misan, Iraq; 8 Research Center for Molecular and Cellular Imaging, Tehran University of Medical Sciences (International Campus), Tehran, Iran; 9 Radiology and Nuclear Medicine Department, School of Paramedical Sciences, Kermanshah University of Medical Sciences, Kermanshah, Iran

**Keywords:** *Duox1*, *Duox2*, Lung, Melatonin, Radiation

## Abstract

**Objective:**

The Lung is one of the most radiosensitive organs of the body. The infiltration of macrophages and lymphocytes into the lung is mediated via the stimulation of T-helper 2 cytokines such as IL-4 and IL-13, which play a key role in the development of fibrosis. It is likely that these cytokines induce chronic oxidative damage and inflammation through the upregulation of *Duox1,* and *Duox2*, which can increase the risk of late effects of ionizing radiation (IR) such as fibrosis and carcinogenesis. In the present study, we aimed to evaluate the possible increase of IL-4 and IL-13 levels, as well as their downstream genes such as *IL4ra1, IL13ra2, Duox1,* and *Duox2*.

**Materials and Methods:**

In this experimental animal study, male rats were divided into 4 groups: i. Control, ii. Melatonin- treated, iii. Radiation, and iv. Melatonin (100 mg/kg) plus radiation. Rats were irradiated with 15 Gy 60Co gamma rays and then sacrificed after 67 days. The expressions of *IL4ra1, IL13ra2, Duox1,* and *Duox2*, as well as the levels of IL-4 and IL-13, were evaluated. The histopathological changes such as the infiltration of inflammatory cells, edema, and fibrosis were also examined. Moreover, the protective effect of melatonin on these parameters was also determined.

**Results:**

Results showed a 1.5-fold increase in the level of IL-4, a 5-fold increase in the expression of IL4ra1, and a 3-fold increase in the expressions of *Duox1,* and *Duox2*. However, results showed no change for IL-13 and no detectable expression of IL13ra2. This was associated with increased infiltration of macrophages, lymphocytes, and mast cells. Melatonin treatment before irradiation completely reversed these changes.

**Conclusion:**

This study has shown the upregulation of IL-4-IL4ra1-Duox2 signaling pathway following lung irradiation. It is possible that melatonin protects against IR-induced lung injury via the downregulation of this pathway and attenuation of inflammatory cells infiltration.

## Introduction

Clinical evidence has shown that more than half of patients with cancers undergo radiotherapy during their course of disease treatment. Normal tissue toxicity is a major limiting factor for tumor control, leading to tumor recurrence and various side effects which affect the quality of life in treated patients (1). Exposure of both normal and tumor cells to ionizing radiation (IR) triggers the production of free radicals such as reactive oxygen species (ROS) and nitric oxide (NO). In addition to direct detrimental effects of IR, these molecules can further amplify damage to cells, resulting in DNA damage and cell death which cause several side effects in the irradiated area (2). The lung is one of the sensitive organs of the human body to the toxic effects of IR. The high radiosensitivity of the lung limits the applied radiation dose for tumor eradication. As non-small cell lung carcinoma (NSCLC) has high resistance to radiotherapy, this results in increased probability of tumor relapses (3). Therefore, to overcome these tumor cells, there is a need for a high dose of IR. However, this may be associated with an increased risk of pneumonitis and fibrosis (4). In recent years, there have been significant improvements in radiotherapy in the delivery of a more precise radiation dose to the tumor volume while sparing normal tissues. However, acute and late normal tissue damage remain an important factor (5). In the lung, radiation-induced side effects such as inflammatory responses (pneumonitis) and fibrosis are the most common limiting factors (6). Currently, there are no appropriate strategies to overcome these complications completely. 

Fibrosis is a process resulting from excessive accumulationof collagen due to differentiation of fibroblasts. It is associatedwith tissue remodeling and affects normal physiologicalfunctions (7). Fibrosis and inflammation in some crucialorgans such as lung, heart, and gastrointestinal system maythreaten patients’ life (8). Experimental and clinical studieshave shown that abnormal increases in the levels of some cytokines such as TGF-ß, IL-1, IL-4, IL-13, TNF-a, etc., havea central role in the development of fibrosis and inflammation(9, 10). The inhibition of some cytokines such as TGF-ß andTNF-a has been most widely studied for the ameliorationof fibrosis and inflammation (11). In addition to TGF-ß, inrecent years, some studies have proposed that IL-4 and IL-13signaling pathways play a key role in the fibrosis process (12). These cytokines trigger the expression of duox1 and duox2through the upregulation of their cognate receptors on cells, which mediate continuous ROS production and stimulationof fibrosis (13). It has been shown that the upregulation ofthese cytokines may induce the infiltration of macrophagesand maintenance of inflammation (14). As pro-oxidantenzymes such as *Duox1, Duox2, NOX1-5, iNOS,* and *COX2* play a key role in continuous ROS production and damageto the normal function of organs, suggesting that targeting ofthese enzymes/genes can help manage normal tissue toxicity during radiotherapy (15). 

Treatment with some adjuvants for sensitization of tumor cells or protection of normal tissue cells is one of the most interesting topics in radiation biology. Melatonin is a natural body hormone that regulates circadian rhythm, as well as several mechanisms in the body such as antioxidant enzyme activity (16). In addition, melatonin has a potent interrelationship with immune system cells (17). In response to radiation, melatonin has shown the ability to protect normal tissues through scavenging of free radicals, stimulation of antioxidant enzymes, as well as anti-inflammatory effects (18, 19). Melatonin has also shown an ability to ameliorate radiation or chemotherapy-induced fibrosis in various organs such as the lung, heart, and others (20). In this study, we examined the effect of pre-treatment with melatonin on the development of fibrosis and histopathological damages following irradiation. Also, we evaluated the possible role of melatonin in alleviating increased levels of IL-4 and IL-13, as well as downstream genes such as *Duox1,* and *Duox2* that are involved in late effects of IR. 

## Materials and Methods

### Experimental design

Melatonin was provided (Merck, Germany) and dissolved in 20% ethanol at a concentration of 20 mg/ ml. 1 ml of the prepared solution (100 mg/kg) was administered to each rat via intraperitoneal injection (18). For this Interventional-experimental study, twenty healthy adult male Wistar rats (200 ± 20 g) were purchased from the Razi Institute, Tehran, Iran. The procedure of this study was in accordance with the ethical laws for animal care provided by Kashan University of Medical Sciences, Kashan, Iran. All animals were housed in suitable conditions, including temperature and humidity (23 ± 2°C and 55%, respectively). They were kept under the same light/dark cycle to prevent any effect of light/dark on basal levels of melatonin in different groups (light 8:00 AM to 8:00 PM and dark 8:00 PM to 8:00 AM). Twenty rats were randomly divided into 4 groups (5 rats in each), group 1: control, group 2: melatonin-treated, group 3: radiation, group 4: melatonintreated+ radiation. Melatonin was administered orally 30 minutes before irradiation. Irradiation was performed using a 60Co source (15 Gy to the whole lung) (21). Sixty-seven days after irradiation, rats were anesthetized, sacrificed, and their lung tissues were extracted. The ventricles were fixed in 10% normal buffer formalin while the auricles were frozen at -80°C for real-time polymerase chain reaction (PCR) and ELISA. 

### Real-time polymerase chain reaction

Total mRNA was isolated from frozen lung tissue of all groups using TRIzol Reagent (GeneAll, South Korea) while cDNA template was synthesized using cDNA synthesis Kits (GeneAll, South Korea) according to the manufacturer’s instructions. *Pgm1* was used as an internal control while the other primers were designed using the Gene runner software and NCBI BLAST tool. Real-time PCR was performed using Corbett’s RT PCR (Qiagen, USA). The primer sequences are shown in Table 1. Real-time PCR efficiency for all mentioned genes in Table 1 was determined using the slope of linear regression as described by Pfaffl (22). Five samples in each group were run in duplicates.

**Table 1:** Forward and reverse primer sequences used in this study


Gene	Sequence primer (5ˊ-3ˊ)

*IL-4r1*	F: GAGTGAGTGGAGTCCCAGCATC
	R: GCTGAAGTAACAGGTCAGGC
*IL- 13ra2*	F: TCGTGTTAGCGGATGGGGAT
	R: GCCTGGAAGCCTGGATCTCTA
*Duox1*	F: AAGAAAGGAAGCATCAACACCC
	R: ACCAGGGCAGTCAGGAAGAT
*Duox2*	F: AGTCTCATTCCTCACCCGGA
	R: GTAACACACACGATGTGGC
*Pgm1*	F: CATGATTCTGGGCAAGCACG
	R: GCCAGTTGGGGTCTCATACAAA


### ELISA

The levels of IL-4 and IL-13 cytokines were detected by the IL-4 and IL-13 ELISA kits (Zellbio, Germany) based on the manufacturer’s instructions. 

### Pathological study

Fixed lung tissues were sectioned at 5-micron sections and then stained with hematoxylin and eosin (H<E) for general tissue characterization. Masson’s trichrome staining (MTC) was also performed for the detection of collagen accumulation. The infiltration of mast cells was evaluated using Giemsa staining. All histopathological studies were performed at the Pathology Unit, Imam Khomeini Hospital, Tehran, Iran, with the aid of a light microscope. 

### Statistical analysis

All statistical analyses were performed using IBM SPSS statistics for windows, version 24.0 (IBM Corp., Armonk, NY, USA). The statistical significance (P<0.05) of mean ± SD for histopathological and ELISA were analyzed using the ANOVAtest followed by post hoc Tukey’s HSD. Furthermore, the expression of genes was analyzed using t test. 

## Results

### ELISA

**Fig.1:**
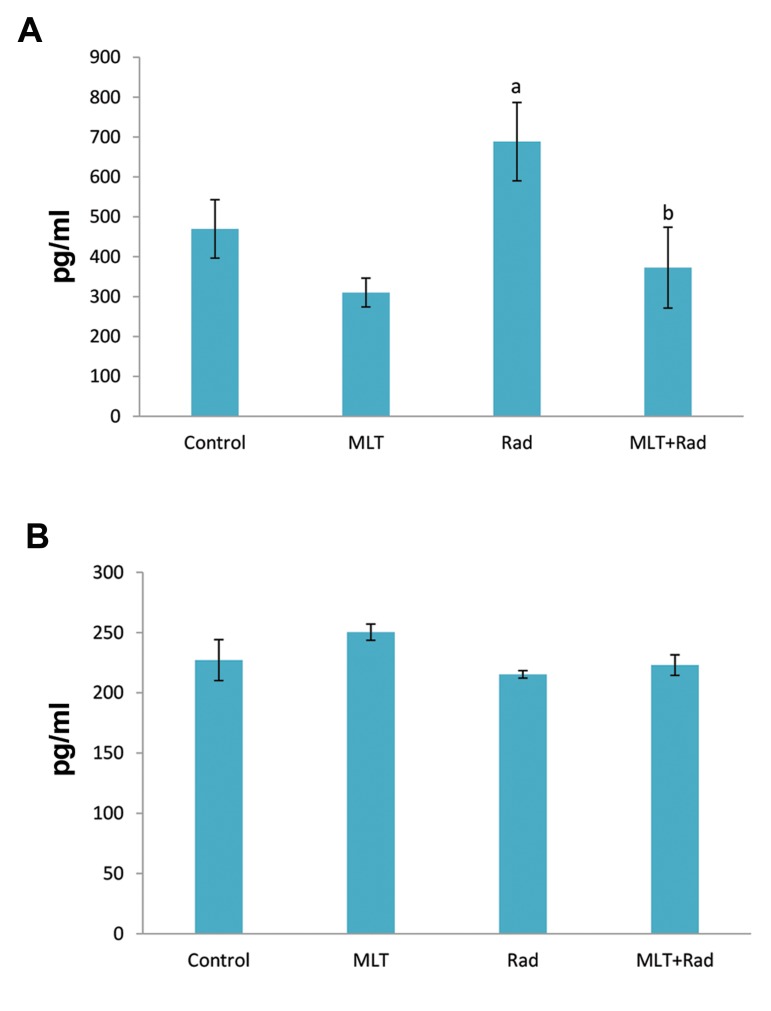
Results of changes in the levels of IL-4 and IL-13 following irradiation with gamma rays and treatment with melatonin (MLT). A. IL-4 and B. IL- 13. a; Significant compared to control and b; Significant compared to radiation (Rad), ANOVA Tukey’s HSD post hoc, P<0.05.

Results showed that irradiation caused a significant increase in the level of IL-4 (702 ± 102) compared to the control group (469 ± 89, P<0.05). Treatment with melatonin before irradiation led to a significant decrease in IL-4 (372 ± 124) compared to the radiation group (P<0.05). Treatment with melatonin alone did not cause any significant change. Results also showed no significant change in the level of IL-13 for all groups (Fig .1). 

### Real-time polymerase chain reaction

**Fig.2:**
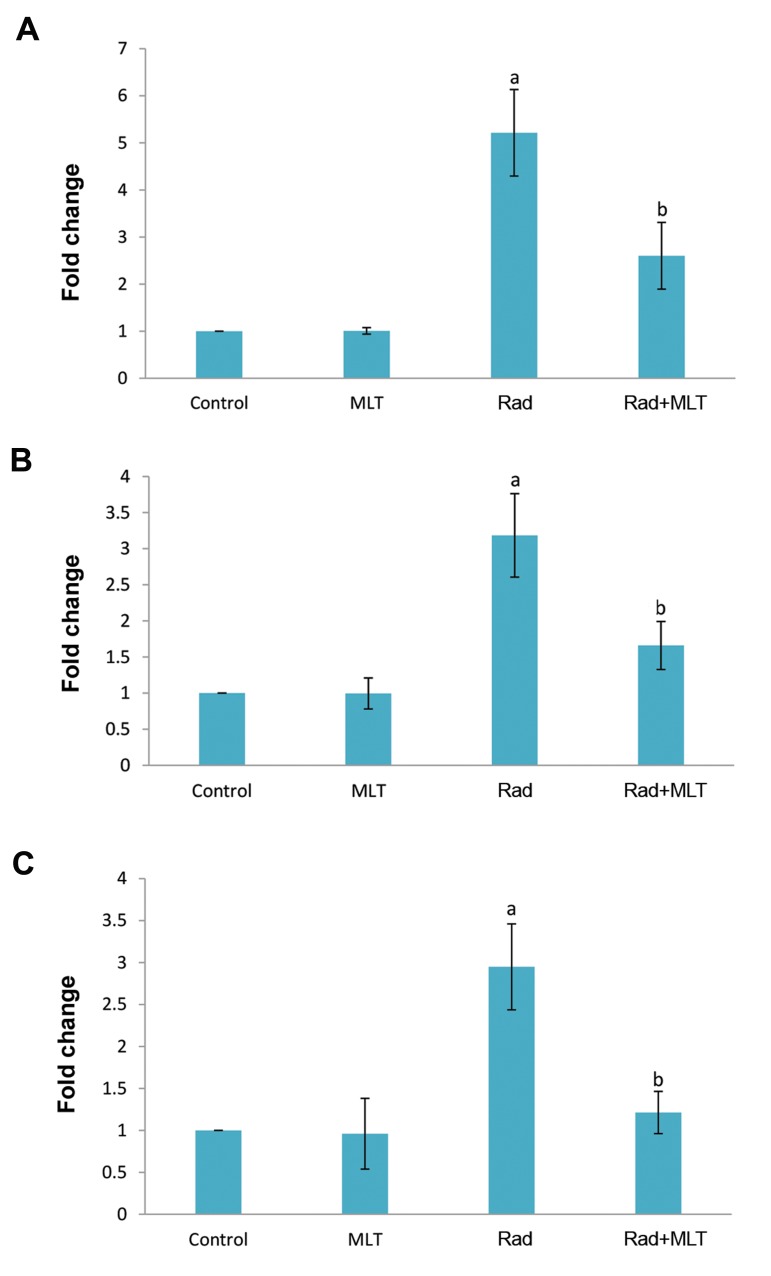
The expression of *IL4ra1, Duox1,* and *Duox2* following irradiation or melatonin treatment before irradiation in lung tissues of rats. **A.** *IL4ra1*, **B.** *Duox1*, and **C.** *Duox2*. a; Significant compared to control and b; Significant compared to radiation (Rad), ANOVA followed by Tukey’s HSD post hoc, P<0.05.

Irradiation of lung tissues was associated with a significant elevation in IL4ra1compared to the control group (5.21 ± 0.92 folds, P<0.05). When rats were treated with melatonin before exposure to IR, the expression of IL4ra1 was significantly decreased compared to the radiation group (2.60 ± 0.70 folds, P<0.05). Results showed no detectable expression for *IL13ra2*. The expression of *Duox1* gene was elevated following exposure to radiation (3.18 ± 0.57 folds, P<0.05). When rats were treated with melatonin before exposure to IR, the expression of *Duox1* was significantly attenuated compared to the radiation group (2.60 ± 0.70, P<0.05). Results of *Duox2* gene expression showed that irradiation caused an increase in its expression in comparison with the control group (2.95 ± 0.51 folds, P<0.001). Treatment with melatonin led to a significant reduction in Duox2 expression (1.21 ± 0.25 folds compared to control) compared to the radiation group (P<0.01, Fig .2). 

### Histopathological assay

Histopathological evaluation showed mild fibrosis in the radiation group. However, this reversed completely when melatonin was administered before irradiation. Also, results showed severe infiltration of macrophages and lymphocytes but not neutrophils. Irradiation led to severe alveolar thickening, as well as mild vascular thickening. Results for edema and thrombosis did not show any significant change. Treatment with melatonin could significantly reverse all these changes (Figes.3-5, Table 2).

**Table 2:** Results of lung irradiation and the protective effect of melatonin


Variable	Control	Melatonin-treated	Radiation	Radiation+Melatonin

Macrophage infiltration	0.25 ± 50	0.25 ± 50	2.66 ± 0.57^a^	0.80 ± 0.83^b^
Lymphocyte infiltration	1.00 ± 0.80	0.50 ± 0.57	3.00 ± 00^a^	0.60 ± 0.54^b^
Mast cell infiltration	0.00 ± 00	1.00 ± 50	4.00 ± 00^a^	3.50 ± 0.50
Neutrophil infiltration	0.50 ± 0.57	0.50 ± 0.57	0.00 ± 00	0.60 ± 0.54
Alveolar thickness	0.25 ± 50	0.25 ± 50	2.00 ± 1.00^a^	0.20 ± 0.44^b^
Vascular thickness	0.00 ± 00	0.00 ± 00	1.00 ± 00^a^	0.00 ± 00^b^
Edema and thrombosis	0.00 ± 00	0.00 ± 00	1.00 ± 0.57	0.00 ± 00
Fibrosis	Absent	Absent	Mild	Absent


Results were scored from 0-3 as 0; Normal, 1; Mild, 2; Severe, 3; Very severe, ^a^; Significant compared to control group, and ^b^; Significant compared to radiation group. Data are presented as mean ± SD.

**Fig.3:**
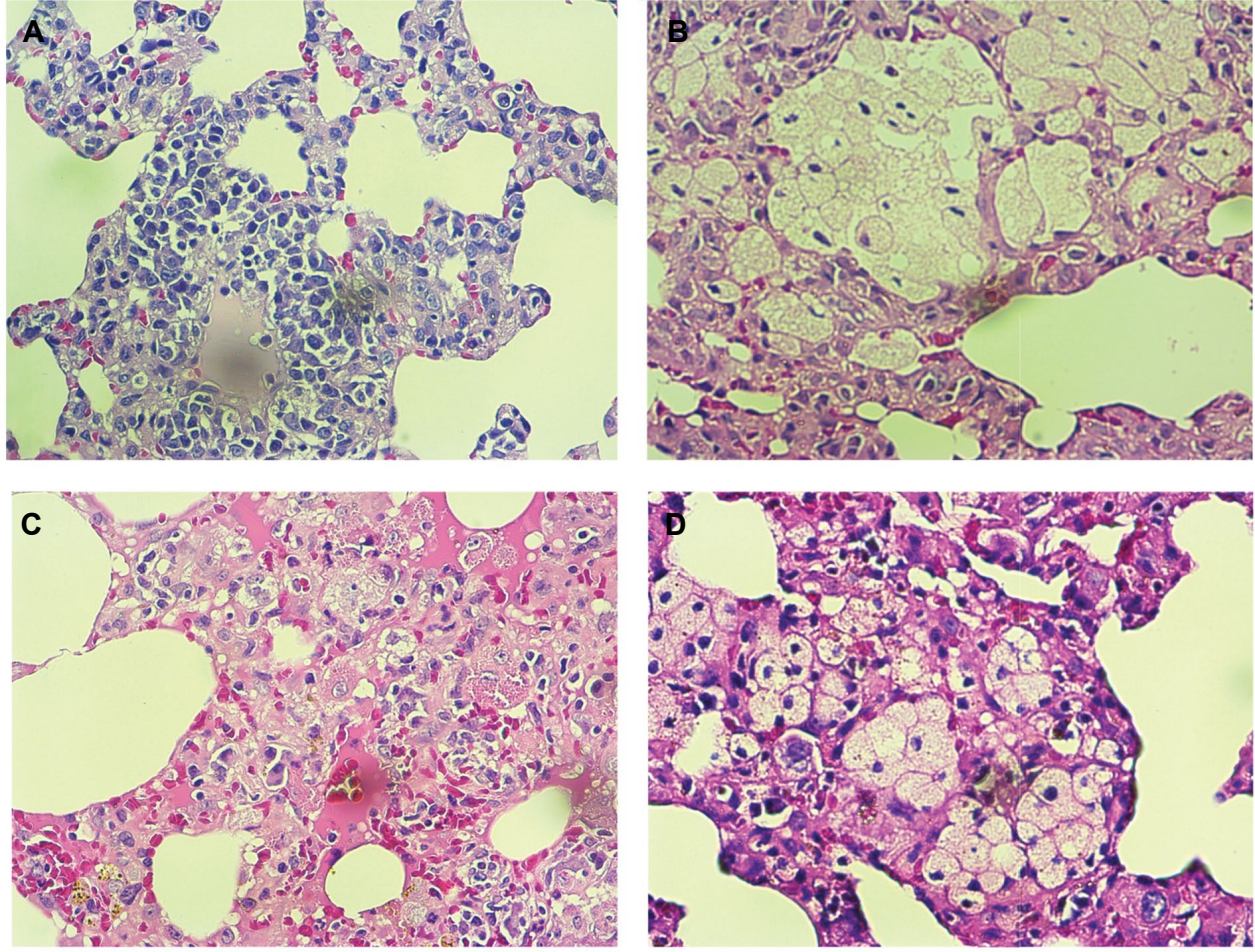
Histopathological investigation of the protective effect of melatonin on radiation-induced lung injury. Control and melatonin groups: no infiltration of macrophages and lymphocytes, as well as normal vascular and alveolar thickening, radiation: severe infiltration of macrophages and lymphocytes, as well as vascular thickening, while alveolar thickening mildly changed. **A.** Control; **B.** Melatonin, **C.** Radiation, **D.** Radiation+Melatonin (H&E staining ×100).

**Fig.4:**
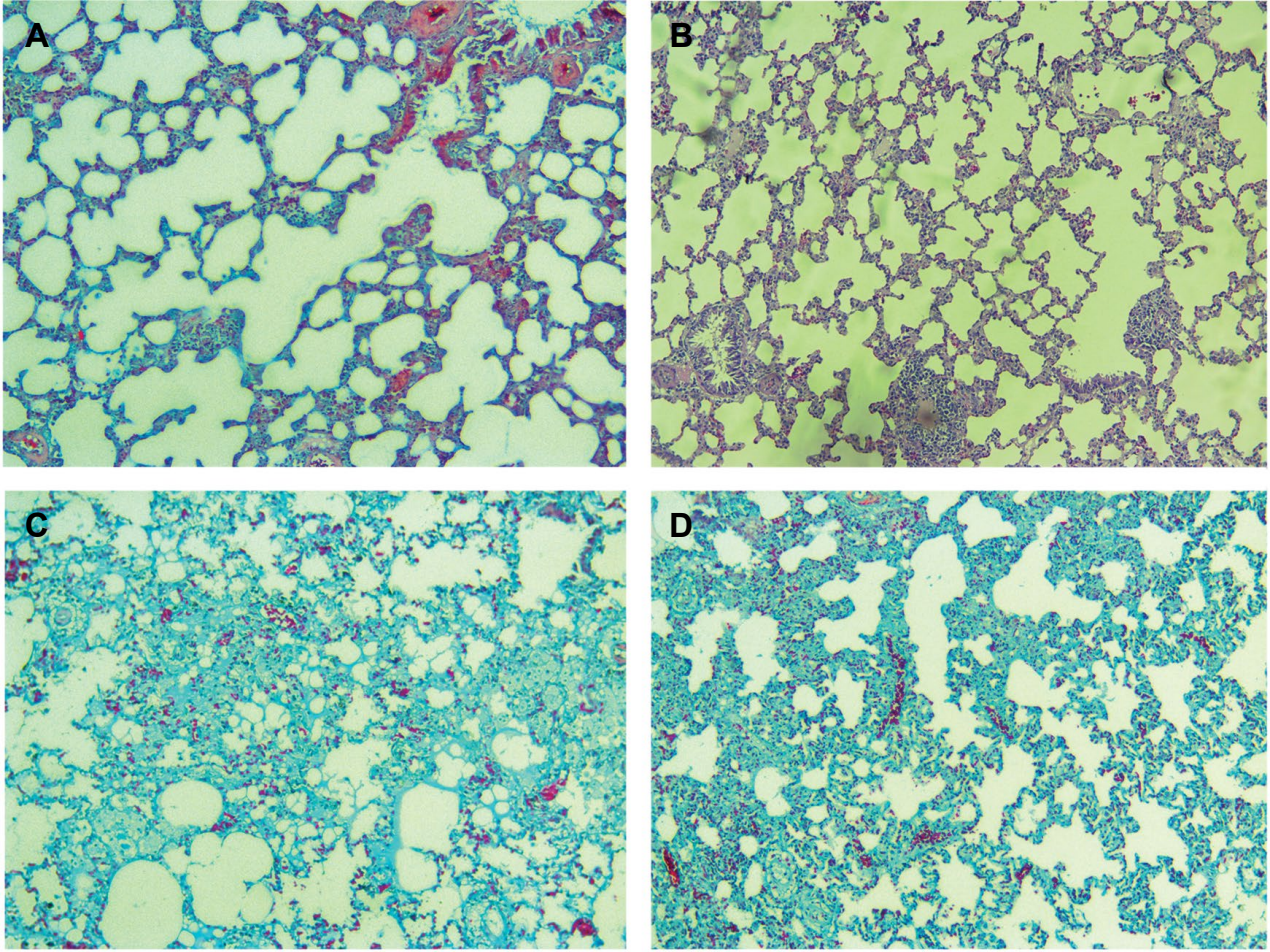
Results of trichrome staining showed a mild collagen deposition, while treatment with melatonin completely reversed collagen deposition. **A.** Control, **B.** Melatonin, **C.** Radiation, and **D.** Radiation+Melatonin (Masson’s Trichrome staining ×100).

**Fig.5:**
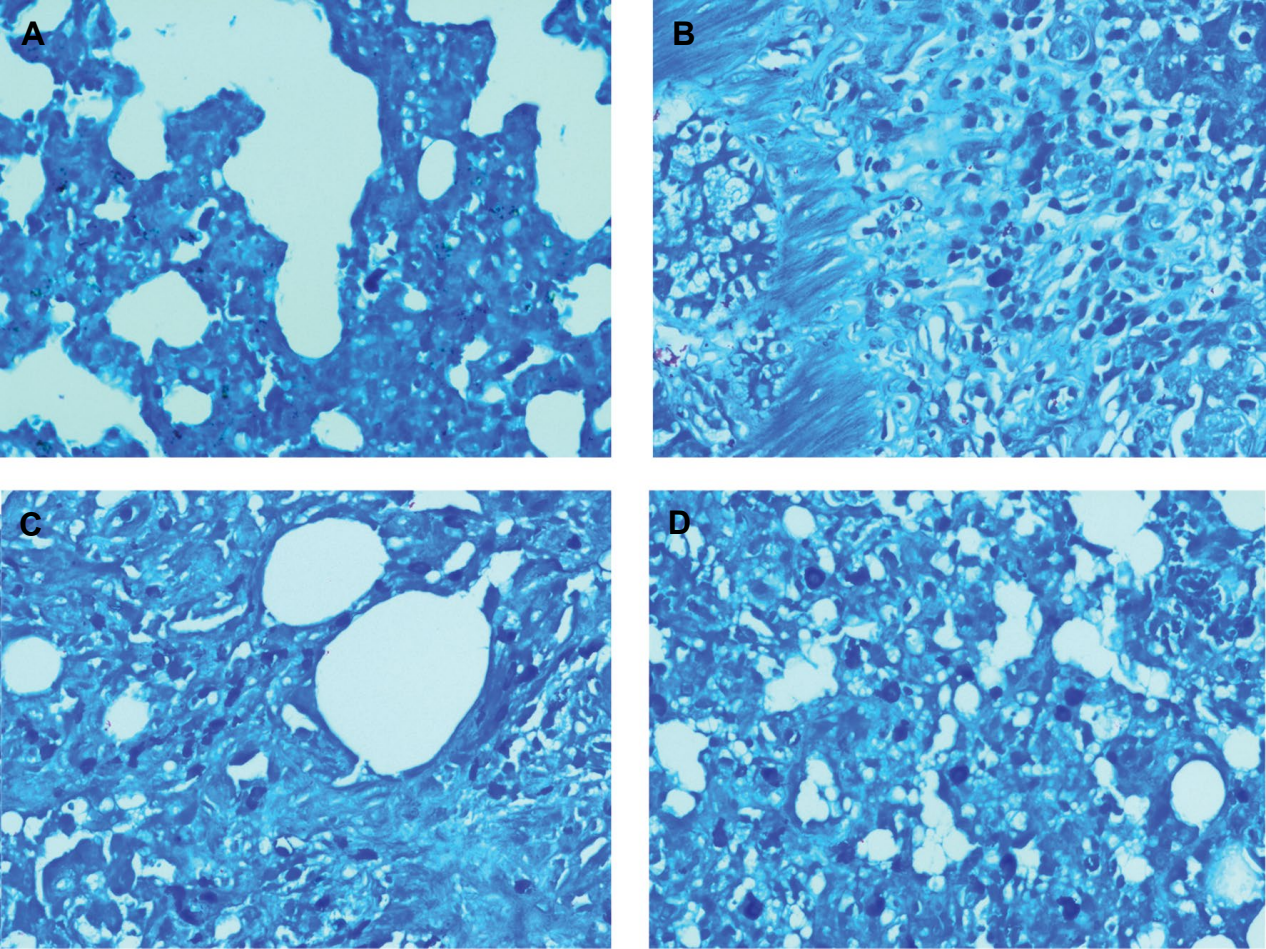
Infiltration of mast cells following irradiation of lung tissues in rats. The administration of melatonin before irradiation could not significantly attenuate mast cell infiltration. **A.** Control, **B.** Melatonin, **C.** Radiation, and **D.** Radiation+Melatonin (Giemsa staining ×100).

## Discussion

The aim of our study was to evaluate changes in the levels of two important pro-fibrotic cytokines and downstream pro-oxidant genes such as *Duox1,* and *Duox2*. Moreover, we detected the possible modulatory effect of melatonin on the changes in the level of these factors. In the present study, we revealed that irradiation of rats’ chest led to a significant increase in the expression of *IL4ra1, Duox1,* and *Duox2* genes. ELISA results showed that the level of IL-4 was increased significantly. 

In contrast to IL-4, results suggest that irradiation of lung tissue did not cause any significant change in the level of IL-13. Moreover, real-time PCR results showed no detectable expression of *IL13ra2*. Probably, the upregulation of *Duox1* was mediated by other signaling pathways, not by *IL13ra2*. A study has shown that, in addition to *IL-13, Duox1* can be upregulated through IL-4 (23). There is a possibility that IL-4 upregulates both *Duox1,* and *Duox2* gene expressions through the stimulation of *IL4ra1*, while IL-13 is not involved in this pathway. There is also a possibility of the involvement of other cytokines such as IFN-γ (24, 25). Our results are in agreement with a study by Groves et al. (26) which showed that IL-4 is involved in the maintenance of macrophages and lung injury following irradiation. However, this study proposed that the development of fibrosis may be induced by other immune mediators but not IL-4. By contrast to our results, Chung et al. (14) reported that after exposure to IR, increased the level of IL-13 but not IL-4 was responsible for the development of lung injuries such as macrophage infiltration and fibrosis. They showed that IL-13 deficiency could reverse lung injury and reduce the expression of genes involved in fibrosis such as TGF-ß, matrix metalloproteinase-2 (MMP-2), and MMP-3. However, this study differed from ours in the sense that they used wild-type c57BL/6NcR mice and a longer time of evaluation. 

As earlier mentioned, the lung is one of the most critical organs for the detrimental effects of IR. It has been reported that the long-term exposure of the lung to radiotherapy due to cancer therapy or iodine therapy for thyroid cancer with metastasis can cause death via pneumonitis or fibrosis (27). In addition to clinical importance for cancer therapy, lung injury may appear following accidental exposure to IR. In this situation, lung late effects may appear following non-uniform whole body exposure or inhalation of radionuclides (28). As the development of lung injury may take a long time to appear, a knowledge of the mechanisms involved in radiation-induced pneumonitis and fibrosis can help better management of them. Although most studies have detected the elevated level of TGF-ß associated with radiation fibrosis, some studies suggest the greater importance of IL-4. It has been proposed that IL-4 plays a central role via the stimulation of other pro-inflammatory and pro-fibrotic cytokines (10). Infiltration of inflammatory cells including macrophages and lymphocytes in irradiated tissues is the source of increased release of IL-4. The accumulation of macrophages and lymphocytes and elevated levels of inflammatory cytokines promote ROS and NO through the stimulation of reduction/oxidation interactions (29). Increased oxidative injury induces a higher degree of inflammation and fibrosis in a positive feedback loop that could finally lead to death (28). 

Histopathological evaluation showed that irradiation of the lung led to severe infiltration of mast cells, macrophages, and lymphocytes, but not neutrophils. This suggests that neutrophil infiltration is involved in late effects of lung injury by IR. Moreover, the histological findings showed mild fibrosis, alveolar, and vascular thickening. Except for mast cell infiltration, all other pathological changes following exposure to IR were alleviated when melatonin was administered 30 minutes before irradiation. Also, melatonin could blunt the upregulation of IL-4 and downstream signaling in IL4ra1 and Duox2. Since macrophages are the main source for the secretion of IL-4 during pneumonitis or fibrosis, it seems that the upregulation of IL-4 signaling after irradiation, as well as the downregulation of that in response to melatonin pretreatment prior to irradiation, is responsible for modulating the infiltration of macrophages. Regarding IL4ra1 and Duox2 can promote continuous ROS production, it is possible that IL-4 signaling plays a key role in radiation-induced fibrosis and vascular injury in the lung. Melatonin treatment before irradiation could reverse the upregulation of these genes completely.

Previous studies have shown that melatonin has the ability to reduce radiation injury via the modulation of several signaling pathways. Melatonin is an FDA- approved drug which has a peak time of absorption up to 40 minutes and a half-life of 1-2 hours (30, 31). Therefore, its administration between 30-60 minutes before exposure to radiation is a common method for radiobiological studies. Melatonin has shown potent anti-inflammatory effects via the prevention of radiation-induced DNA damage and cell death (32). Melatonin can also elevate DNA repair capacity to mitigate cell death (19). By means of the inhibition of Toll-like receptors (TLRs), transcription factors, pro-oxidant enzymes, as well as pro-fibrotic and pro-inflammatory cytokines melatonin attenuates redox activity and relieves late effects of IR (20, 33). As a result of these properties, melatonin is appropriate for radiation countermeasure, protection, and mitigation of lung injury by IR (34).

## Conclusion

Results of this study showed that irradiation of rats’lung led to a significant increase in the level of IL-4 and pro- oxidant genes such as *Duox1,* and *Duox2*. However, we did not observe any significant increase in the level of IL13, as well as the expression of IL13ra2. This could be an indication that radiation induces lung inflammation and fibrosis via the upregulation of IL-4 but not IL-13. This suggests that the infiltration of macrophages plays a key role in the stimulation of IL-4 and its downstream genes. In addition, we showed that melatonin administration before irradiation reversed the infiltration of macrophages and lymphocytes, as well as the upregulation of *IL-4, IL4ra1, Duox1,* and *Duox2*.
